# Mitogenic Effects of Phosphatidylcholine Nanoparticles on MCF-7 Breast Cancer Cells

**DOI:** 10.1155/2014/687037

**Published:** 2014-03-20

**Authors:** Yamila B. Gándola, Sebastián E. Pérez, Pablo E. Irene, Ana I. Sotelo, Johanna G. Miquet, Gerardo R. Corradi, Adriana M. Carlucci, Lorena Gonzalez

**Affiliations:** ^1^Instituto de Química y Fisicoquímica Biológicas (UBA-CONICET), Facultad de Farmacia y Bioquímica, Junín 956, 1113 Buenos Aires, Argentina; ^2^Departamento de Tecnología Farmacéutica, Facultad de Farmacia y Bioquímica, Junín 956, 1113 Buenos Aires, Argentina

## Abstract

Lecithins, mainly composed of the phospholipids phosphatidylcholines (PC), have many different uses in the pharmaceutical and clinical field. PC are involved in structural and biological functions as membrane trafficking processes and cellular signaling. Considering the increasing applications of lecithin-based nanosystems for the delivery of therapeutic agents, the aim of the present work was to determine the effects of phosphatidylcholine nanoparticles over breast cancer cellular proliferation and signaling. PC dispersions at 0.01 and 0.1% (w/v) prepared in buffer pH 7.0 and 5.0 were studied in the MCF-7 breast cancer cell line. Neutral 0.1% PC-derived nanoparticles induced the activation of the MEK-ERK1/2 pathway, increased cell viability and induced a 1.2 fold raise in proliferation. These biological effects correlated with the increase of epidermal growth factor receptor (EGFR) content and its altered cellular localization. Results suggest that nanoparticles derived from PC dispersion prepared in buffer pH 7.0 may induce physicochemical changes in the plasma membrane of cancer cells which may affect EGFR cellular localization and/or activity, increasing activation of the MEK-ERK1/2 pathway and inducing proliferation. Results from the present study suggest that possible biological effects of delivery systems based on lecithin nanoparticles should be taken into account in pharmaceutical formulation design.

## 1. Introduction

Lecithins are a mixture of phospholipids where phosphatidylcholines are the main components (up to 98% w/w). Egg or soy lecithin as well as purified phospholipids is used for pharmaceutical purposes as dispersing, emulsifying, and stabilizing agents included in intramuscular and intravenous injectables or parenteral nutrition [1–3]. Lecithins have been used to form liposomes, mixed micelles, and submicron emulsions for pharmaceutical purposes. Moreover, aqueous lecithin dispersions (water-lecithin-dispersion (WLD)) alone or in combination with cationic molecules have been proposed as carriers of lipophilic drugs and even as oligonucleotides delivery systems for cancer treatment [4, 5]. Actually, nanoparticles designed from lecithin-in-water emulsions were successfully used to deliver docetaxel to tumor cells* in vitro* and even in a tumor model in mice [6]. Moreover, lecithin-based nanoparticles have demonstrated to deliver siRNA to breast cancer cells [7].

Phosphatidylcholines, the main components of lecithins, are glycerophospholipids that incorporate choline as the head group. The fatty acids bound to the glycerophosphatidic acid can vary but generally one of them is unsaturated and the other one is saturated. Phosphatidylcholine (PC) is a major constituent of the cell membranes which is more commonly found in the exoplasmic or outer leaflet of the plasma membrane. PC also plays a role in membrane-mediated cell signaling. The phospholipase D-mediated catabolism of PC yields phosphatidic acid (PA) and choline, which are important lipid second messengers involved in several signaling pathways [8–10]. PA binds to Raf-1 and promotes its recruitment to the plasma membrane where it is activated by direct interaction with Ras [11, 12]. Ras-mediated Raf-1 activation leads to mitogen-activated protein kinase (MAPK) and PI3K/Akt activation [13]. Therefore, PA would have a pivotal role in the amplification of signaling cascades required for survival and growth [14]. PA also binds the mammalian target of rapamycin (mTOR), a protein kinase that regulates cell cycle progression and cell growth regulating several cellular events like translation, transcription, membrane trafficking, and protein degradation [15].

Phosphatidylcholine is also a substrate of the phosphatidylcholine-specific phospholipase C (PC-PLC). This enzyme has been implicated in proliferation, differentiation, and apoptosis of mammalian cells. PC-PLC-mediated hydrolysis of PC yields PC-derived diacylglycerol (DAG) and phosphocholine (P-chol) [8, 16]. DAG and P-chol, in turn, activate a variety of kinases involved in cell proliferation, including MAPKs, in different cell types [17, 18].

The lipid second messengers PA and DAG that are generated as a result of PLD and PC-PLC activity, respectively, can also affect membrane trafficking, directly by altering membrane curvature or indirectly by recruiting and/or activating signaling mediators [19]. PLD-derived PA has been linked to vesicular trafficking processes including Golgi transport, endocytosis, and exocytosis [19]. Moreover, aberrant phosphatidylcholine metabolism in cancer cells was reported to downmodulate the membrane expression of specific receptors or proteins relevant for cell proliferation and survival [20, 21]. Particularly, inhibition of phosphatidylcholine-specific phospholipase C downregulates Human Epidermal Growth Factor Receptor 2 (HER2) overexpression on plasma membrane of breast cancer cells [21]. Likewise, membrane phospholipid composition was demonstrated to affect epidermal growth factor receptor (EGFR) endocytosis [22]. Lipid composition not only affects EGFR trafficking but also has relevant regulatory effects on its kinase domain activation and signaling [22, 23].

Membrane phospholipids as well as their fatty acid profile are altered in tumor cells. The choline metabolite profile of cancer cells is characterized by an elevation of phosphocholine and total choline-containing compounds. Indeed, total cellular phosphatidylcholine (PC) can be used as a marker for membrane proliferation in neoplastic mammary gland tissues [24] or as a predictive biomarker for monitoring tumor response [25].

Phosphatidylcholines are therefore not inert vehicles but biological active compounds; phospholipids and their derived second messengers are involved in cell proliferation and trafficking, and the increase of phosphocholine and choline-containing compounds has been described in tumor cells. It has been recently highlighted that certain excipients have a role as active pharmaceutical components of formulations because they can modify the pharmacological activity of an active drug or produce biological effects [26]. Considering that phosphatidylcholines are the main components of lecithins and taking into account the increasing applications of lecithin-based formulations in nanomedicine and for the delivery of antineoplastic agents, the aim of the present work was to determine the biological effects of phosphatidylcholine nanoparticles over breast cancer cell signaling and proliferation.

## 2. Material and Methods

### 2.1. Reagents

Purified phosphatidylcholine from soybean lecithin (Phospholipon 90G, CAS-number 97281-47-5) was purchased from Lipoid (Ludwigshafen, Germany). Trizma base, HEPES, Tween 20, Triton X-100, sodium dodecyl sulfate (SDS), glycine, ammonium persulfate, aprotinin, phenylmethylsulfonyl fluoride (PMSF), sodium orthovanadate, 2-mercaptoethanol, Hoechst 33258, and BSA-fraction V were obtained from Sigma Chemical Co. (St. Louis, MO, USA). PVDF membranes, high performance chemiluminescence film, and enhanced chemiluminescence- (ECL-) Plus are from Amersham Biosciences (GE Healthcare, Piscataway, NY, USA). Mini-Protean apparatus for SDS-polyacrylamide electrophoresis, miniature transfer apparatus, acrylamide, bis-acrylamide, and TEMED were obtained from Bio-Rad Laboratories (Hercules, CA, USA). Anti-EGFR (1005) antibody and secondary antibodies conjugated with HRP were purchased from Santa Cruz Biotechnology Laboratories (Santa Cruz, CA, USA). Antibodies anti-phospho-mTOR Ser2448, anti-mTOR, anti-p44/42 MAP kinase (ERK 1/2), and anti-phospho-p44/42 MAP kinase Thr202/Tyr204 were from Cell Signaling Technology Inc. (Beverly, MA, USA). Cy3-conjugated secondary antibody against rabbit polyclonal immunoglobulins was from Jackson ImmunoResearch Laboratories, Inc. Bicinchoninic acid (BCA) protein assay kit was obtained from Thermo Scientific, Pierce Protein Research Products (Rockford, IL, USA).

### 2.2. Preparation of Phosphatidylcholine Nanoparticles

Dispersions of Phospholipon 90G 0.01 and 0.1% (w/v) in two different diluents (66 mM isotonic phosphate buffer pH 7.0 and 50 mM isotonic acetate buffer pH 5.0) were prepared. Buffers were isotonized by adding sodium chloride when necessary according to Sörensen and White-Vincent methods. Phosphatidylcholine was first dispersed in the appropriate diluent with means of extensive mixing at 60°C by use of a thermostated magnetic stirrer in order to obtain good hydration. Next, the dispersion was stirred for 2 minutes at the same temperature with a high-shear mixer (Ultra-Turrax T18 basic, IKA Werke, Staufen, Germany) and sonicated at 20 kHz for 10 minutes. It was then sterilized by autoclaving (121°C, 15 minutes). The sizes of the resulting particles in the dispersions were determined by photon correlation spectroscopy (PCS) using a Zetasizer (Malvern Nano ZS, Malvern Instruments Ltd., UK). The zeta potential of the samples was measured by the same instrument and the zeta potential values were calculated according to Smoluchowski equation (Table 1). As shown in Table 1, particles in the range of the nanometric size were obtained.

### 2.3. Cell Culture

MCF-7 human breast cancer cell line was obtained from the American Type Culture Collection (ATCC) (Rockville, MD, USA). Cells were maintained in Dulbecco's minimum essential medium (DMEM) supplemented with 10% fetal bovine serum (FBS), 50 *μ*g/mL gentamycine (Invitrogen, Life technology), and 2 mM L-glutamine (Invitrogen, Life technology). Cells were cultured in 75 cm^2^ culture flasks at 37°C in a humidified atmosphere of 5% CO_2_.

### 2.4. Culture Cells Treatment

To perform immunoblotting assays, cells were seeded in clear 6-well plates (Corning Costar, Fisher Scientific, USA) at a density of 300,000 cells/well, while for immunofluorescence assays cells were seeded at a density of 20,000 cells/well in covers placed in 24-well plates. Phosphatidylcholine nanoparticles at 0.1 and 0.01% were added in the presence or absence of serum. Cells were further incubated at 37°C for 24 hours in a 5% CO_2_ atmosphere. After incubation, cells were washed with phosphate saline buffer and dishes were kept at −80°C until cell solubilization to prepare cells extracts, while covers were immediately processed for specific immunofluorescence labeling.

### 2.5. Preparation of Cell Extracts and Immunoblotting

Cells were homogenized in buffer composed of 1% v/v Triton, 0.1 M Hepes, 0.1 M sodium pyrophosphate, 0.1 M sodium fluoride, 0.01 M EDTA, 0.01 M sodium vanadate, 0.002 M PMSF, and 0.035 trypsin inhibitory units/mL aprotinin (pH 7.0) at 4°C. Cell homogenates were centrifuged at 15,000 ×g for 40 minutes at 4°C to remove insoluble material. Protein concentration of supernatants was determined by the BCA protein assay kit. Equal protein aliquots of solubilized cells were diluted in Laemmli buffer, boiled for 5 minutes, and stored at −20°C until electrophoresis.

Samples were subjected to electrophoresis in SDS-polyacrylamide gels. Electrotransference of proteins from gel to PVDF membranes and incubation with antibodies were performed as already described [27]. Immunoreactive proteins were revealed by enhanced chemiluminescence. Band intensities were quantified using Gel-Pro Analyzer 4.0 software (Media Cybernetics, Silver Spring, MD, USA).

To reprobe with other antibodies, the membranes were washed with acetonitrile for 10 minutes and then incubated in stripping buffer (2% w/v SDS, 0.100 M 2-mercaptoethanol, 0.0625 M Tris/HCl, pH 6.7) for 40 minutes at 50°C while shaking, washed with deionized water, and blocked with BSA.

### 2.6. Cell Viability Assay

Cells were seeded in clear 96-well plates (Corning Costar, Fisher Scientific, USA) at a density of 10,000 cells/well. Phosphatidylcholine at 0.1 and 0.01% was added in 100 *μ*L of medium in the presence or absence of serum. Cells were further incubated at 37°C for 24 and 48 hours in a 5% CO_2_ atmosphere. After incubation with the phosphatidylcholine nanoparticles, cell number was evaluated using the CellTiter 96 aqueous nonradioactive cell proliferation assay (Promega, Madison, WI, USA). Triplicates were run for each treatment. Values were expressed in terms of percent of untreated control cells.

### 2.7. BrdU Incorporation Assay

DNA synthesis in proliferating cells was determined by measuring BrdU incorporation with a BrdU ELISA assay [28]. For this purpose, the cells were seeded in 96-well culture plates at a density of 10,000 cells/well. Phosphatidylcholine nanoparticles at 0.1 and 0.01% were added in 100 *μ*L of medium in absence of serum. Cells were further incubated at 37°C for 48 hours in a 5% CO_2_ atmosphere. BrdU (0.01 M final concentration) was added to the cells 16 hs before the end of incubation with PC nanoparticles. At the conclusion of labeling, cultures were rinsed with phosphate buffered saline (PBS), pH 7.0, fixed with 70% EtOH, denatured with 2 M HCl (100 *μ*L/well, 10 minutes, 37°C), and neutralized with 0.1 M Trizma buffer, pH 9. Cells were then incubated with monoclonal anti-BrdU antibody (50 *μ*L/well; 1 *μ*g/mL final; Roche, USA) at 37°C for 60 minutes, washed with PBS, and incubated with goat anti-mouse IgG horseradish peroxidase (HRP) conjugate at 37°C for 30 minutes. Afterwards, cells were washed and labeling evidenced with tetramethylbenzidine (TMB). Triplicates were run for each treatment. Values were expressed in terms of percent of untreated control cells.

### 2.8. Immunofluorescence

Cells were washed twice in PBS, pH 7.0, fixed in 2% formaldehyde in PBS for 10 minutes at room temperature. After three washes with PBS (5 minutes each), fixed cells were permeabilized with 0.5% Triton X-100 in PBS for 15 minutes and incubated in blocking solution (10% FBS in PBS) for 30 minutes to decrease nonspecific binding of the antibodies. Cells were then incubated for 1 hour at 37°C with anti-EGFR, then washed, and incubated with Cy3-conjugated secondary antibody against rabbit polyclonal immunoglobulins. After a final washing step (3 washes 5 minutes each in PBS), cells were incubated with Hoechst 33258 (2 *μ*g/mL) for ten minutes. Finally, covers were mounted on glass slides and fluorescence stained cells were imaged by epifluorescent microscopy on a Leica DM2000 with a ×40 objective (Numerical Aperture =0.65) or by an Olympus Fluoview FV1000 spectral laser scanning confocal microscope with a ×60 oil immersion objective (Numerical Aperture =1.35) using dual excitation (473 nm for Cy3 and 405 nm for Hoesch). At least 10 fields were examined and representative images were photographed.

### 2.9. Statistical Analysis

Experiments were performed analyzing the phosphatidylcholine dispersions and vehicle (control) in parallel, *n* representing the number of different experiments. Results are presented as mean ± S.E.M. Statistical analyses were performed by ANOVA followed by the Newman-Keuls Multiple Comparison Test using the GraphPad Prism 4 statistical program by GraphPad Software, Inc. (San Diego, CA, USA). Data were considered significantly different if *P* < 0.05.

## 3. Results 

### 3.1. Phosphatidylcholine Nanoparticles Activate Cell Signaling Molecules Involved in Cell Proliferation

Previous results from our research group have demonstrated that nanoparticles prepared from phosphatidylcholine dispersed at 0.01 and 0.1% (w/v) in buffer pH 5.0 and buffer pH 7.0 are able to bind oligonucleotides and deliver them to breast cancer cells [7]. To determine oligonucleotide internalization, transfection experiments were performed either in absence or presence of serum and after a 24-hour incubation period [7]. That study suggested that lecithin-based delivery systems might represent feasible novel formulations for anticancer gene therapies. However, phosphatidylcholines, the main components of lecithin, are involved in several biological processes like cell proliferation and dynamics of the cell membrane. To ascertain if PC nanoparticles have* per se* promitogenic activity, the effects of phosphatidylcholine-based nanoparticles over signal transduction pathways involved in cell proliferation and survival were studied in the previously described experimental conditions [7]. Considering that PC-derived second messengers are involved in the activation of cellular signaling mediators like mTOR and MAPKs [9, 29, 30], activation of the Akt-mTOR and MEK1/2-ERK1/2 signaling pathways by phosphatidylcholine was analyzed in the MCF-7 breast cancer cell line.

#### 3.1.1. Akt and mTOR Phosphorylation and Protein Content

**Figure 1:**
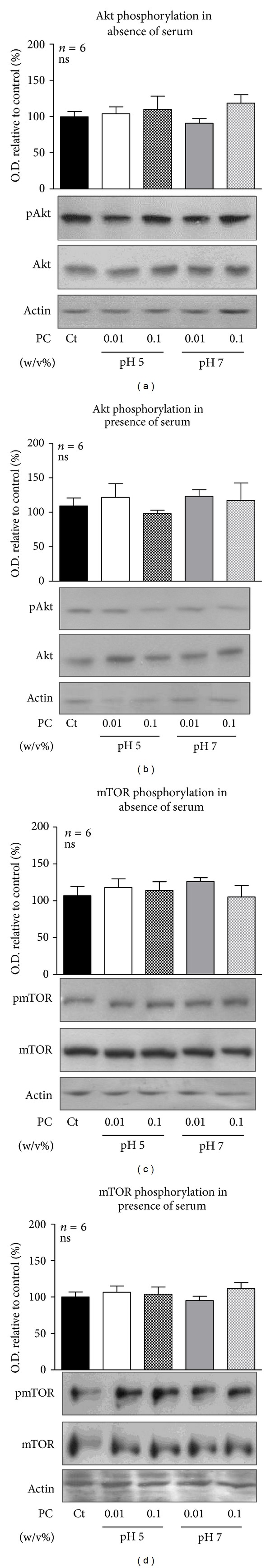
Akt and mTOR phosphorylation and protein content. MCF-7 breast cancer cells were incubated for 24 hs with PC nanoparticles dispersed at 0.1 and 0.01% (w/v) in buffer pH 5.0 and buffer pH 7.0 or vehicle (Ct) in the absence ((a) and (c)) or presence ((b) and (d)) of serum. Representative results of immunoblots with anti-Akt and anti-phospho-Akt S473 ((a) and (b)) and anti-mTOR and anti-phospho-mTOR S2448 ((c) and (d)) are shown. Reprobing with anti-actin antibody demonstrated uniformity of protein loading in all lanes. Quantification of phosphorylated proteins was performed by scanning densitometry and expressed as percent of values measured for control, nonstimulated breast cancer cells (Ct). Data are expressed as the mean ± S.E.M. of the indicated number (*n*) of different experiments. Statistical analysis was performed by ANOVA.

Akt is activated by many types of cellular stimuli and regulates fundamental cellular functions such as transcription, translation, proliferation, growth, and survival. Its dysregulation has been associated with the development of diseases such as cancer [31, 32]. Akt phosphorylation and protein content of MCF-7 cells previously treated with aqueous phosphatidylcholine dispersions were analyzed by western blotting. Akt phosphorylation at Ser473, an activating residue, was not statistically different in control and treated cells neither in absence (Figure 1(a)) nor in presence (Figure 1(b)) of serum. Incubation with phosphatidylcholine had no effects on Akt protein content from MCF-7 cells (Figures 1(a) and 1(b)).

Mammalian target of rapamycin complex is a Ser/Thr kinase of the phosphatidylinositol 3-kinase-related kinase protein family. Akt phosphorylates and activates mTOR, thus inducing protein synthesis and cell growth [15, 33]. mTOR activation and protein content were studied by western blotting in cells treated with phosphatidylcholine nanoparticles (Figures 1(c) and 1(d)). Resembling the results obtained for Akt, mTOR phosphorylation was not induced by PC either in absence or in presence of serum, even when cells were incubated with the highest concentrations of phosphatidylcholine dispersions (Figures 1(c) and 1(d)).

#### 3.1.2. MEK and ERK 1/2 Phosphorylation and Protein Content

The Ras/Raf/MEK/ERK cascade couples signals from cell surface receptors to transcription factors, which can regulate cell cycle progression, apoptosis, or differentiation [34]. This signaling cascade is often activated in certain tumors by chromosomal translocations, mutations in cytokine receptors, or overexpression of wild type or mutated receptors.

MAP kinase kinase (MEK) is a dual-specificity kinase that phosphorylates tyrosine and threonine residues on extracellular-signal-regulated kinases 1 and 2 (ERK 1/2) [35]. Two related genes encode MEK1 and MEK2. Under basal conditions, MEK binds the inactive serine/threonine kinase ERK and restricts it to the cytosol. The MEK/ERK complex dissociates when MEK is activated and phosphorylates ERK, which may then dimerize. An activated ERK dimer can regulate targets in the cytosol and also translocate to the nucleus where it phosphorylates a variety of transcription factors regulating gene expression.

**Figure 2:**
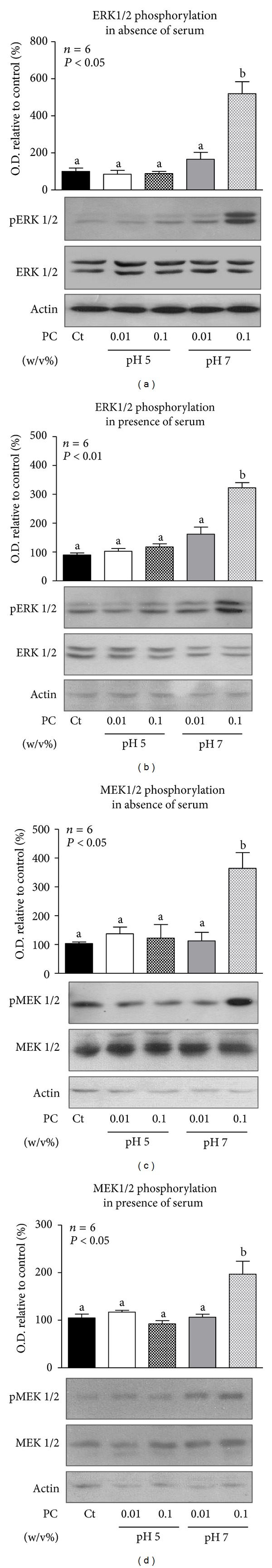
ERK1/2 and MEK1/2 phosphorylation and protein content. MCF-7 breast cancer cells were incubated for 24 hs with PC nanoparticles dispersed at 0.01 and 0.01% (w/v) in buffer pH 5.0 and buffer pH 7.0 or vehicle (Ct) in absence ((a) and (c)) or presence ((b) and (d)) of serum. Representative results of immunoblots with anti-p44/42 MAP kinase (ERK1/2) and anti-phospho-p44/42 MAP kinase Thr202/Tyr204 ((a) and (b)) and anti-MEK1/2 and antiphospho MEK ((c) and (d)) are shown. Reprobing with anti-actin antibody demonstrated uniformity of protein loading in all lanes. Quantification of phosphorylated proteins was performed by scanning densitometry and expressed as percent of values measured for control, nonstimulated breast cancer cells (Ct). Data are expressed as the mean ± S.E.M. of the indicated number (*n*) of different experiments. Statistical analysis was performed by ANOVA. Different letters denote significant difference at *P* < 0.05, whereas results with the same letter are not statistically different from each other.

Phosphorylation of MEK1/2 and ERK1/2 was studied in the MCF-7 cells incubated with PC nanoparticles. Results showed that MEK1/2 and ERK1/2 phosphorylation was significantly increased when cells were treated with phosphatidylcholine dispersed in pH 7.0 solution at high concentration independently of the absence (Figures 2(a) and 2(c)) or presence (Figures 2(b) and 2(d)) of serum. PC nanoparticles dispersed in buffer pH 7.0 at low concentration (0.01%) showed a slight tendency to stimulate ERK1/2 phosphorylation; however, this difference did not achieve statistical significance (Figures 2(a) and 2(b)). MEK1/2 and ERK1/2 protein levels did not vary either in absence or in presence of serum.

### 3.2. Phosphatidylcholine Nanoparticles Induce MCF-7 Cell Proliferation

**Figure 3:**
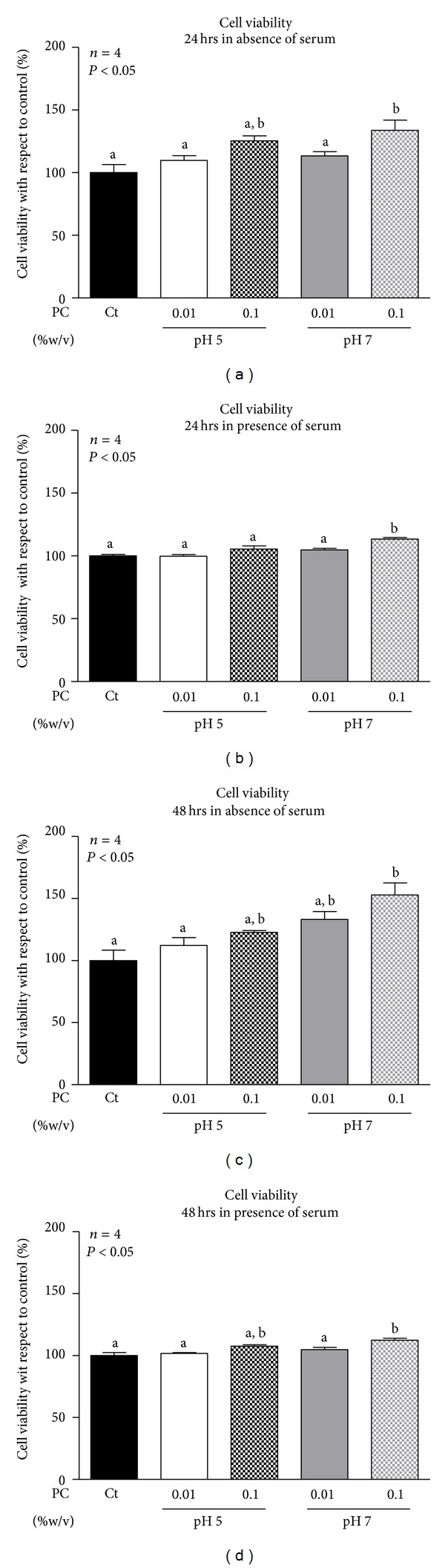
Viability of MCF-7 breast cancer cells incubated with phosphatidylcholine (PC) nanoparticles. Breast cancer cells were incubated for 24 hours ((a) and (b)) and 48 hours ((c) and (d)) with PC nanoparticles dispersed at 0.01 and 0.1% (w/v) or vehicle (Ct) in buffer pH 5.0 and buffer pH 7.0 in the absence ((a) and (c)) or the presence ((b) and (d)) of serum. After incubation, cell viability was evaluated using the CellTiter 96 aqueous nonradioactive cell proliferation assay (Promega). Triplicates were run for each treatment. Values were expressed in terms of percent of untreated control cells set as 100%. Data are expressed as the mean ± S.E.M. of the indicated number (*n*) of independent experiments. Statistical analysis was performed by ANOVA. Different letters denote significant difference at *P* < 0.05, whereas results with the same letter are not statistically different from each other.

As it was previously mentioned, MEK1/2-ERK 1/2 signaling pathway is involved in cell growth and proliferation promotion, so the effects of phosphatidylcholine nanoparticles over breast cancer cell viability were studied. For this purpose, MCF-7 cells were seeded in 96-well plates and incubated during 24 hours (Figures 3(a) and 3(b)) or 48 hours (Figures 3(c) and 3(d)) with phosphatidylcholine dispersed at 0.1 and 0.01% in the absence (Figures 3(a) and 3(c)) or presence (Figures 3(b) and 3(d)) of serum. Results showed that only high concentration of PC nanoparticles dispersed in buffer pH 7.0 significantly increased cell viability of MCF-7 breast cancer cells either in absence (Figures 3(a) and 3(c)) or presence of serum (Figures 3(b) and 3(d)) at both time periods. Phosphatidylcholine dispersed in buffer pH 5.0 or in buffer pH 7.0 at low concentration (0.01%) had moderate effects on cell viability but results were not statistically significant (Figure 3).

**Figure 4:**
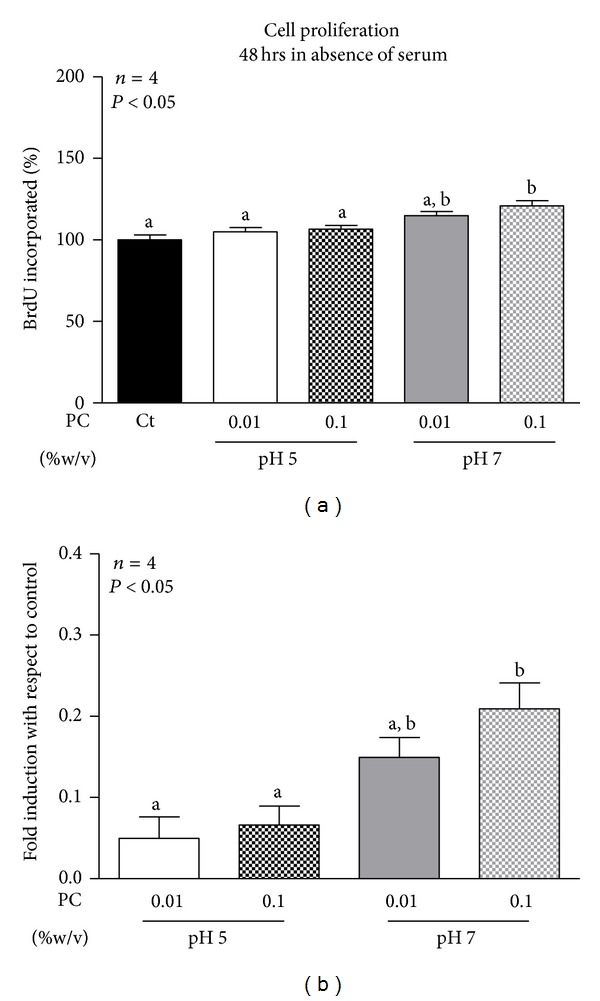
Proliferation of MCF-7 breast cancer cells incubated with phosphatidylcholine (PC) nanoparticles. Breast cancer cells were incubated for 48 hours with PC nanoparticle prepared in buffer pH 7.0 at 0.01 and 0.1% (w/v) or vehicle (Ct) in the absence of serum (a). Proliferation was determined by measuring BrdU incorporation with a BrdU ELISA. Triplicates were run for each treatment. Values were expressed in terms of percent of untreated control cells set as 100% (a). Data are expressed as the mean ± S.E.M. of the indicated number (*n*) of independent experiments. Fold induction with respect to control was calculated (b). Statistical analysis was performed by ANOVA. Different letters denote significant difference at *P* < 0.05, whereas results with the same letter are not statistically different from each other.

To ascertain if the increased cell viability induced by phosphatidylcholine nanoparticles 0.1% at pH 7.0 was a consequence of cell proliferation induction, BrdU incorporation assay was performed. Considering that the differences between viability of basal cells and PC-treated cells were better evidenced when cells were treated in the medium without serum, BrdU incorporation was assessed after 48 hours of treatment with phosphatidylcholine nanoparticles in absence of serum. Results demonstrated that increased cell viability correlated with increased incorporation of BrdU (Figure 4). When fold induction of BrdU incorporation was calculated it was observed that PC 0.1% at pH 7.0 induces a 20% increment in cell proliferation (Figure 4(b)).

The effects of phosphatidylcholine 0.1% dispersed in buffer pH 7.0 over MCF-7 cell proliferation correlated with the increased phosphorylation levels observed for MEK 1/2 and ERK1/2. Results suggest that high concentration of phosphatidylcholine nanoparticles at pH 7.0 induces activation of the MEK1/2-ERK1/2 pathway and cell proliferation of the breast cancer cells.

### 3.3. EGFR Levels Are Increased in Breast Cancer Cells Treated with High Concentration of Phosphatidylcholine Nanoparticles

Molecular aspects of cell signaling are controlled by receptor/ligand localization and trafficking [36, 37]. Endocytosis and subsequent delivery of endosomal cargos to lysosomes are essential for the degradation of many membrane-associated proteins [38–40]. This process determines the amplitude of growth factor signaling, and it is therefore tightly regulated.

As previously mentioned, PC and second messengers derived from these phospholipids are fundamental components of the cell membrane and affect its dynamics and protein trafficking. Particularly, previous studies have demonstrated that phospholipid membrane composition affects EGF receptor endocytosis and signaling [22, 23]. When purified EGFR was reconstituted into proteoliposomes of specific lipid compositions, the lipid environment did not affect EGF binding but EGFR tyrosine kinase function was indeed modified [23]. Moreover, mutants in the Drosophila phosphocholine cytidylyltransferase 1 (CCT1), the rate-limiting enzyme in PC biosynthesis, result in altered phospholipid composition of cell membranes and affect the endocytic pathway of EGFR [22].

Endosomal trafficking of EGFR is crucial for determining the amplitude and duration of EGFR signaling. Actually, endocytosis of the EGFR is required for EGF-induced MAP kinase activation. This was evidenced in experiments in which EGF induction of MAPKs was reduced in dynamin mutant cells which showed defects in clathrin-dependent receptor-mediated endocytosis [41]. Treatment of breast cancer cells with high concentration of phosphatidylcholine nanoparticles could affect membrane composition and consequently trafficking of EGFR and signaling through MAPKs. Therefore, EGFR levels were determined by western blotting and EGFR cellular localization was studied by immunofluorescence of cells treated with the PC nanoparticles.

**Figure 5:**
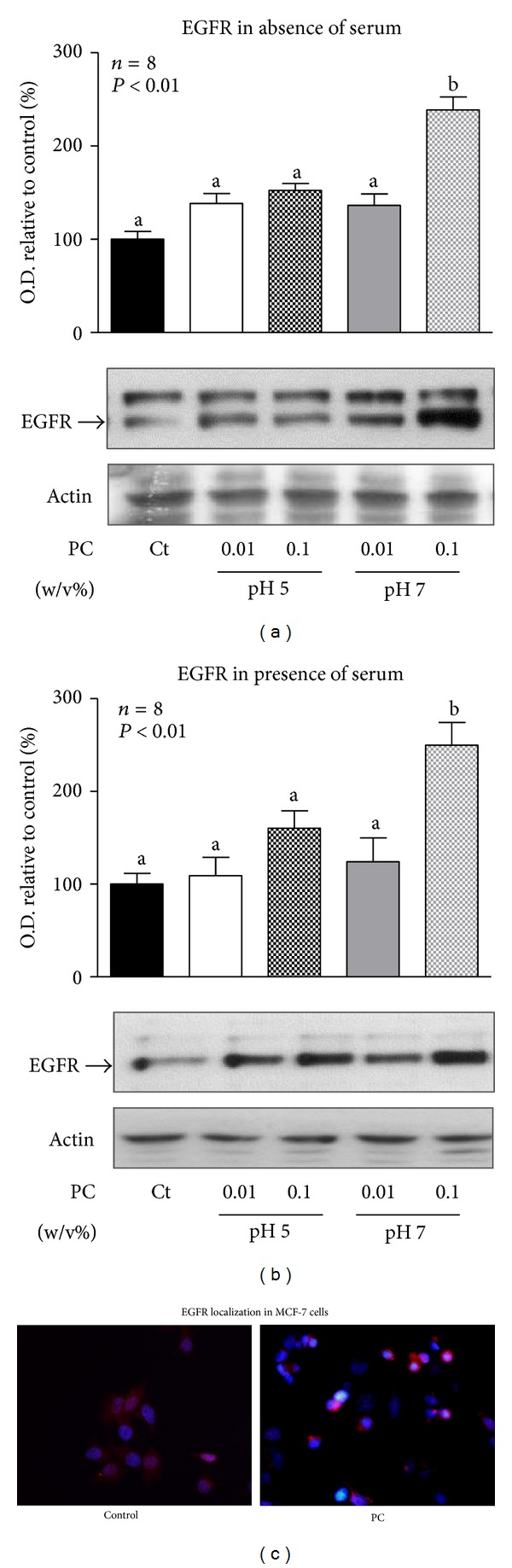
EGFR protein content and immunocytochemistry. MCF-7 breast cancer cells were incubated for 24 hours with PC nanoparticles dispersed at 0.1 and 0.01% (w/v) in buffer pH 5.0 and buffer pH 7.0 or vehicle (Ct) in absence (a) or presence (b) of serum for immunoblot studies, but only with PC dispersions at 0.1%, pH 7.0, or vehicle (control) in absence of serum for immunocytochemistry (c). Western blotting was performed as described in M & M. Membranes were reprobed to asses actin content and demonstrate equal protein loading in all lanes. Representative immunoblots are shown ((a) and (b)). EGFR quantification was performed by scanning densitometry and expressed as percent of values measured for control, nonstimulated breast cancer cells. Data are expressed as the mean ± S.E.M. of the indicated number (*n*) of independent experiments. Statistical analysis was performed by ANOVA. Different letters denote significant difference at *P* < 0.05, whereas results with the same letter are not statistically different from each other. For EGFR immunocytochemistry (c), cells were washed, fixed, permeabilized, blocked, and incubated with the anti-EGFR antibody, the Cy3-conjugated secondary antibody, and Hoechst. Finally covers were mounted and examined by epifluorescence microscopy. Representative merged images of control and PC-nanoparticles-treated cells are shown (c).

**Figure 6:**
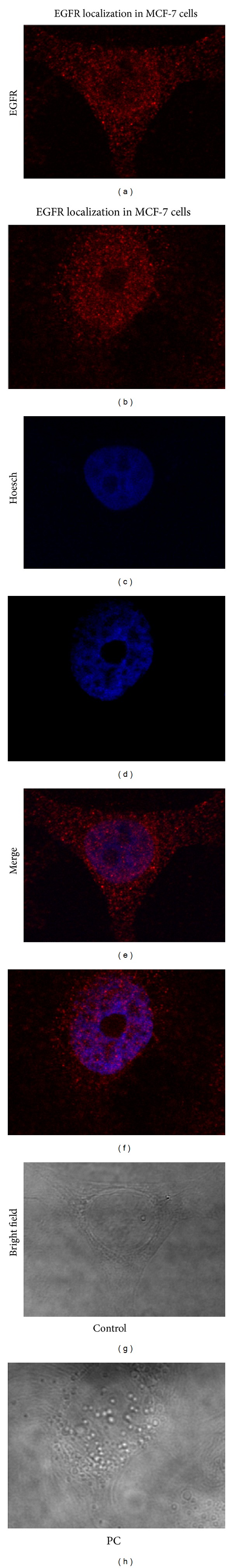
EGFR immunocytochemistry. MCF-7 breast cancer cells were incubated for 24 hours with PC nanoparticles dispersed at 0.1 in buffer pH 7.0 or vehicle (Ct) in absence of serum. For EGFR immunocytochemistry, cells were washed, fixed, permeabilized, blocked, and incubated with the anti-EGFR antibody, the Cy3-conjugated secondary antibody, and Hoechst. Finally covers were mounted and examined by confocal microscopy. Images were obtained using sequential scanning. Representative images of EGFR, nuclear staining, merge, and bright field are shown.

Results showed that EGFR levels increased when cells were treated with PC nanoparticles dispersed in buffer pH 7.0 at high concentration both in absence or presence of serum (Figures 5(a) and 5(b)). Afterwards, immunofluorescence studies were conducted to ascertain the cellular localization of increased EGFR in cells treated with PC 0.1% in pH 7.0. For comparison, EGFR-immunofluorescence was performed in control and phosphatidylcholine-treated cells. Observation of stained cells by epifluorescence microscopy showed that EGFR was uniformly distributed in control cells, while a significant proportion of PC-treated cells showed an increase in perinuclear EGFR staining (Figure 5(c)). However, PC-treated cells exhibited an increased percentage of rounded cells which might account for an increased proliferation rate. These morphological changes could explain the pattern of EGFR labeling observed when analyzed by epifluorescent microscopy. Therefore, confocal microscopy studies were performed to analyze possible alterations in EGFR cellular localization produced by treatment with PC nanoparticles (Figure 6). According to images obtained by epifluorescent microscopy, a significant proportion of PC-treated cells showed increased nuclear and perinuclear distribution of the EGFR (Figure 6).

## 4. Discussion

Despite the multiple and different uses of lecithin with pharmaceutical and therapeutic purposes, the possible biological consequences of phosphatidylcholine administration should be considered. They are important phospholipids involved not only in structural functions in the cell but also in membrane trafficking processes and signaling. Moreover, increased levels of phosphocholine and choline-containing compounds have been associated with progression and bad prognosis of tumors. Considering the increasing use of lecithin-based formulations for the delivery of antineoplastic agents, the biological effects of nanoparticles derived from aqueous phosphatidylcholine dispersions over breast cancer cells proliferation and signaling were studied. Previously characterized phosphatidylcholine nanoparticles proposed as oligonucleotide delivery systems were used for that purpose [7]. Results showed that PC nanoparticles prepared in neutral buffer induced the activation of the MEK-ERK1/2 pathway and increased cell viability and proliferation of the MCF-7 breast cancer cell line. In accordance, Erk1/2 activation by phosphatidylcholine liposomes has been described to mediate neuronal differentiation [42, 43].

Incubation with the phosphatidylcholine nanoparticles prepared in neutral buffer was associated with increased EGFR content in the cancer cells and with its altered cellular localization. High phosphatidylcholine concentrations might induce physicochemical changes in the plasma membrane that affect receptor trafficking and turnover. Moreover, a process has been recently described, dependent on sustained stimulation of cPCK and PLD activities, that leads to EGFR sequestration near the perinuclear region, in the pericentrion [44]. Accumulation of EGFR, reflected by increased EGFR content and perinuclear localization of the receptor, would result in increased activation of ERK1/2 and increased cell proliferation. Besides interfering with EGFR trafficking, PC nanoparticles might also have effects over EGFR activity [22, 23, 45]. Ligand-independent dimerization of EGFR occurs with reasonable frequency; however, it is not activated until the binding of the ligand. An autoinhibitory mechanism involving the EGFR C-terminal tail would explain the lack of activity of the dimer [45]. Interaction of phosphatidylcholine nanoparticles with inactive EGFR dimers could be proposed as a possible mechanism that disables such regulatory mechanisms and leads to EGFR activation even in absence of the specific ligand. Moreover, activation of the dimer involves reorganization of hydrophobic regions of the EGFR [46]; nanoparticles obtained from dispersed PC at buffer pH 7.0 might favor such reorganization facilitating the formation of the active conformation.

Increased concentration of phosphatidylcholine nanoparticles dispersed in buffer pH 7.0 had significant effects over cell proliferation, EGFR levels, and activation of the MEK1/2-ERK1/2 pathways; however, such effects were not observed for PC nanoparticles dispersed in pH 5.0 buffer. The main differences between both PC preparations is the charge associated with the particles (zeta potential) (Table 1) and the final adopted form [7], while the size of the particles did not show important differences (Table 1). The nanoparticles were in the range of 180–250 nm for all the studied conditions (Table 1). At pH 5.0, small, isolated particles with irregular shape were observed while at pH 7.0 more elongated, locally cylindrical structures were described [7]. As expected, the zeta potential of the particles was positive when using pH 5.0 buffer as diluent and negative when using pH 7.0 buffer (Table 1). This fact can be related to changes in the proportion of the differently charged forms of the zwitterionic phosphocholine polar head within the selected pH range and the conformational organization the molecules acquire as a result.

In spite of the studies accomplished using lipid-based nanocarriers for drug and gene delivery, the relationship between their physicochemical characteristics and activation of membrane receptors remains as an area of knowledge with incipient development. In this regard, cationic liposomal lipids have been described to modify cellular pathways and stimulate immune or anti-inflammatory responses [47]. However, the mechanisms responsible for those biological effects are poorly understood. Contrary to previous reports concerning cationic lipids, the present study shows that biological effects are induced when cells are incubated with the negatively charged phosphatidylcholine nanoparticles but not when positively charged PC nanoparticles are administrated. Nevertheless, the mechanisms involved could be similar to that proposed for cationic lipids; insertion of a negatively charged phospholipid-derived nanoparticle in the biological membrane might modify the lipid environment of membrane proteins, the lipid-protein interaction and, therefore, membrane functioning.

## 5. Conclusion

Results from the present study suggest that high phosphatidylcholine concentrations, assembled in negatively charged nanoparticles, may induce physicochemical changes in the plasma membrane that affect EGFR cellular localization and/or its activity, therefore facilitating accumulation of the receptor in the cytoplasm, which would be associated with increased activation of the MEK-ERK1/2 pathway and induction of cell cycle progression. It is interesting that the described effects were specifically observed for the phosphatidylcholine nanoparticles prepared in pH 7 buffer but not at pH 5; so we propose that this might be related to the different net charge and morphology associated with the particles, as no significant differences in size between nanoparticles obtained from dispersion at pH 7.0 and 5.0 were observed. Considering that the PC nanoparticles preformed in a pH 5.0 buffer showed no significant biological effects over the breast cancer cells, these would be safer than those prepared in a pH 7.0 buffer to deliver antimitotic agents.

The interpretation of the interaction between nanocarriers with membrane receptors is a matter that must be elucidated for a more appropriate understanding of the biological effects that are promoted. The present study highlights the importance of the research on the effects of vehicles broadly used in the pharmaceutical area and demonstrates that possible biological effects of formulations based on phosphatidylcholine nanoparticles should be considered. Moreover, studies about the possible biological action of PC nanoparticles on normal cells would be useful to expand our knowledge about their potential pharmaceutical uses. Excipient effects over normal physiology and cell biology represent important factors to be concerned about in rational formulation design.

## Figures and Tables

**Table 1:** Effect of pH on the particle size and zeta potential of the phosphatidylcholine nanoparticles.

Formulation	Particle size (*d*.nm) ± SD	PdI	Z-Pot (mV) ± SD
pH 5.0	232.7 ± 19.6	0.494	13.8 ± 2.1
pH 7.0	189.1 ± 11.9	0.544	−40.3 ± 3.0

*d*.nm: diameter expressed in nm; PdI: polidispersion index.

Phosphatidylcholine (PC) nanoparticles were prepared in pH 5.0 and pH 7.0 buffers and analyzed by Dynamic Light Scattering (DLS). The size and zeta potential of the particles were measured and reported as mean ± S.E.M. (*n* = 4).
